# Proteomics analysis reveals age-related proteins in the urine of chronic kidney disease patients

**DOI:** 10.3389/fmed.2024.1506134

**Published:** 2025-01-06

**Authors:** Lin Xiong, Changwei Wu, Sipei Chen, Yong Zhang, Li Wang, Yi Li, Guisen Li

**Affiliations:** ^1^Department of Nephrology and Institute of Nephrology, Sichuan Provincial People’s Hospital, School of Medicine, University of Electronic Science and Technology of China, Sichuan Clinical Research Center for Kidney Diseases, Chengdu, China; ^2^Institutes for Systems Genetics, West China Hospital, Sichuan University, Chengdu, China

**Keywords:** LC–MS/MS, urine proteomics, aging, biomarker, chronic kidney disease

## Abstract

Chronic kidney disease (CKD) is closely linked to the aging process, making the identification of protein biomarkers that reflect aging in specific organs and tissues crucial for a deeper understanding of this phenomenon. This study aimed to identify potential aging-related proteins present in the urine of CKD patients. Utilizing liquid chromatography–tandem mass spectrometry (LC–MS/MS) proteomic analysis, we identified a total of 1,712 proteins in the urine samples from both healthy controls and CKD patients in our discovery cohort. Among the 845 proteins that overlapped, we found that 161 proteins were associated with aging. By applying a threshold of *p* < 0.05 and |log2 (fold change) | > 1.5, we classified 114 proteins as differentially expressed proteins (DEPs). The analyzes conducted using the Gene Ontology and the Kyoto Encyclopedia of Genes and Genomes revealed that DEPs were significantly enriched in several clusters related to aging. In the validation cohort, we demonstrated that patients with CKD exhibited lower urinary levels of L-selectin (SELL), uromodulin (UMOD), and epidermal growth factor (EGF). Additionally, a significant negative correlation was found between age and EGF levels. The estimated glomerular filtration rate (eGFR) showed a significant positive correlation with SELL, UMOD, and EGF, while 24-h proteinuria showed a significant negative correlation with both UMOD and EGF. Furthermore, both UMOD and EGF were significantly negatively correlated with tubulointerstitial fibrosis, and EGF was significantly negatively correlated with glomerulosclerosis. In conclusion, this study emphasizes the promise of LC–MS/MS-based urine proteomics analysis in identifying aging-related protein markers. Specifically, SELL, UMOD, and EGF have been recognized as promising indicators of aging in patients with CKD.

## 1 Introduction

Chronic kidney disease (CKD) represents a significant public health challenge globally, with a prevalence rate of 9.1% reported in 2017 (1). In addition, patients with CKD face markedly higher mortality across all stages of the disease (2). It is estimated that CKD will become the fifth most common cause of death in the world by 2040 (3). As individuals age, significant structural and functional changes occur in the kidneys, including a decrease in kidney cortical volume, glomerulosclerosis, tubulointerstitial fibrosis, and a decrease in estimated glomerular filtration rate (eGFR) (4). Compelling evidence indicates that CKD plays a substantial role in accelerating the aging process, particularly affecting the immune system, as well as skeletal and cardiovascular aging (5–7). In patients with end-stage kidney disease (ESKD) undergoing hemodialysis, a high fracture risk has been identified as an independent predictor of all-cause mortality (8). Furthermore, calciphylaxis, a serious and potentially life-threatening vascular condition, has a prevalence exceeding 1.24% among Chinese hemodialysis patients (9). Therefore, it is reasonable to conclude that CKD contributes to a reduced lifespan, closely linked to accelerated aging.

Aging is a complex and multifactorial process characterized by a systematic and progressive decline in biological functions (10). Despite extensive research and the formulation of numerous aging theories, achieving a comprehensive understanding of the aging process remains a significant challenge (11). This difficulty can be partly attributed to the heterogeneity of aging, as the characteristics and rates of decline can vary both between different organs and within the same organ (12). Therefore, it is crucial to identify specific aging markers that can help characterize the aging process across various organs and tissues.

Liquid chromatography–tandem mass spectrometry (LC–MS/MS)-based proteomics has recently garnered significant attention, paving the way for innovative approaches to analyze and identify aging markers (13–15). Urine has emerged as one of the most promising biofluids in clinical proteomics, offering several advantages such as easy accessibility, straightforward handling, relative stability, and few risk of damages (16). Notably, urinary proteins are not solely secreted by the kidneys and urinary tract; they also include proteins from distant organs and tissues that are filtered from plasma through the glomeruli (17). Consequently, we hypothesize that urine proteomics could yield insights into both systemic and local organ aging. However, to our knowledge, there is currently limited understanding of aging markers present in urine, particularly those characterizing the aging status of specific organs and tissues.

The objective of this study was to identify aging-related protein biomarkers in the urine of patients with CKD through LC–MS/MS-based urine proteomics. To begin, we integrated a range of protein markers that characterize specific organ and tissue aging, as reported by Oh’s team and the National Institutes of Health Cellular Senescence Network (SenNet) Consortium (18, 19). Ultimately, we examined and confirmed the correlation between urinary aging-related proteins and clinical parameters indicative of renal function and aging.

## 2 Materials and methods

### 2.1 Study participants and study design

In this study, a total of 239 participants were recruited from 2019 to 2023 at the Sichuan Provincial People’s Hospital. These participants were divided into a discovery cohort (*n* = 157) and a validation cohort (*n* = 82). The discovery cohort comprized 35 patients with diabetic kidney disease (DKD), 50 patients with IgA nephropathy (IgAN), and 72 healthy subjects. The validation cohort included 20 patients with DKD, 31 patients with IgAN, and 31 healthy subjects. Patients diagnosed with DKD or primary IgAN were included in the study. The exclusion criteria primarily encompassed the presence of comorbid cancers, severe infections, and malnutrition.

All healthy subjects were considered healthy and were utilized as healthy controls (HC) group. Given that DKD (20) and IgAN (21) are the two most prevalent causes of CKD, we combined patients with DKD or IgAN into a CKD group. Additionally, we gathered general demographic, clinical, and pathological parameters of the patients within this group, including the estimated Glomerular Filtration Rate (eGFR) calculated using the CKD-EPI equation (22). One subject from the discovery cohort was excluded due to significant missing data. This study was performed in accordance with the Helsinki Declaration and was approved by the Institutional Ethics Committee of Sichuan People’s Hospital (No. 2024.109). Written informed consent was obtained from all enrolled subjects.

### 2.2 Urinary protein digestion and mass spectrometric analysis

In the discovery cohort, urine was collected within a week prior to renal biopsy, centrifuged at 1000 g for 20 min, and then 500 μL of the supernatant was stored at −80°C until analyzed by LC–MS/MS. The detailed methods of proteomics, including urinary protein digestion, mass spectrometer analysis, and spectral establishment, have been described in our previous reports (23). Briefly, urinary protein digestion was performed using a filter-aided sample preparation. Urine peptide analysis was conducted on Orbitrap Fusion Lumos mass spectrometer (Thermo Fisher Scientific, Waltham, MA, USA). The RAW data from the mass spectrometer were converted into MGF format files, and the mass spectra from each file are used to urine proteomics profile of each subject. The mass spectrometry proteomics data from the urine proteomics have been deposited to the ProteomeXchange Consortium via the PRIDE partner repository with the dataset identifier PXD018996.

### 2.3 Principal component analysis and orthogonal partial least squares discriminant analysis

To investigate the protein segregation trends between HC group and CKD group, the unsupervised principal component analysis (PCA) and supervised orthogonal partial least squares discriminant analysis (OPLS-DA) were performed on the free online platform of the Majorbio Cloud (https://cloud.metware.cn) (24).

### 2.4 Differentially expressed proteins and functional enrichment analysis

In this study, proteins with |log_2_ (fold change) | >1.5 and *p* < 0.05 were defined as differentially expressed proteins (DEPs) (25). The Gene Ontology (GO) and Kyoto Encyclopedia of Genes and Genomes (KEGG) analyzes of DEPs were performed using the Xiantao Academic (Xiantao, China) (https://www.xiantaozi.com). Significant GO items and pathways were defined for an adjusted *p*-value of <0.05.

### 2.5 Enzyme-linked immunosorbent assay

In the validation cohort, urine was detected by Enzyme-linked immunosorbent assay (ELISA). The human plasma protease C1 inhibitor (SERPING1) ELISA kit (Lot No. ZC-57270), human L-selectin (SELL) ELISA kit (Lot No. ZC-31978), human uromodulin (UMOD) ELISA kit (Lot No. ZC-34290) and human epidermal growth factor (EGF) ELISA kit (Lot No. ZC-32514) were purchased from Shanghai zcibio technology Co., Ltd. (Shanghai, China). All assays were performed in accordance with the manufacturer’s instructions.

### 2.6 Statistical analysis

All statistical analyzes were performed using IBM SPSS Statistics 25.0 (SPSS Inc., Chicago, IL, USA). The normality of the data was assessed using the Shapiro–Wilk test. Data that followed a normal distribution were expressed as mea*n* ± standard deviation (m ± s). Differences between two groups were analyzed using t-tests, and differences between multiple groups were compared using one-way ANOVA, followed by the Bonferroni test for post-hoc comparison. Non-normally distributed data were presented as median with interquartile range [IQR], with the Mann–Whitney U test used to compare two groups and the Kruskal-Wallis H test for multiple groups. Categorical data were presented as frequencies (percentages) and compared using the chi-square test. Correlation analyzes were performed using Spearman’s correlation test. All *p*-values were two-sided, and *p* < 0.05 was considered statistical significance.

## 3 Results

### 3.1 Demographic and baseline characteristics of the participants

In the discovery cohort, the CKD group exhibited a higher proportion of males compared to HC group (56% vs. 31%, *p* = 0.001). The median age for both the CKD and HC groups is comparable, with both being 45 years old. There were no notable differences between the discovery and validation cohorts regarding gender, age, the prevalence of hypertension and diabetes, blood triglyceride levels, blood urea nitrogen (BUN), serum creatinine (Scr), eGFR, 24-h proteinuria, glomerulosclerosis, and tubulointerstitial fibrosis (Table 1).

**Table 1:** Demographic and clinical parameters of patients with CKD in the discovery cohort and validation cohort.

Parameters	Discovery cohort (*n* = 84)	Validation cohort (*n* = 51)	*P*-value
Gender (male, %)	47 (56%) ^c^	26 (51%) ^c^	0.574
Age (year)	45 (31, 54) ^b^	45 (35, 52) ^b^	0.801
Hypertension (%)	52/84 (62%) ^c^	36/51 (53%) ^c^	0.305
Diabetes mellitus (%)	34/84 (41%) ^c^	21/51 (41%) ^c^	0.936
Triglyceride (mmol/L)	1.8 (1.33, 2.56) ^b^	2.06 (1.31, 3.36) ^b^	0.534
Hemoglobin (g/L)	124.23 ± 22.36 ^a^	123.57 ± 21.2 ^a^	0.866
Blood urea nitrogen (mmol/L)	7.43 (5.39, 10.47) ^b^	6.9 (5.23, 9.58) ^b^	0.739
Serum creatinine (μmol/L)	110.35 (72.03, 145.85) ^b^	117.2 (74.5, 153.2) ^b^	0.533
eGFR (mL/min)	61.8 (43.01, 101.5) ^b^	55.7 (41.88, 89.69) ^b^	0.399
Proteinuria (g/24 h)	3.05 (1.38, 5.03) ^b^	1.72 (0.97, 4.34) ^b^	0.054
Glomerulosclerosis (%)	17.42 (8.52, 27.27) ^b^	16.67 (5.88, 33.33) ^b^	0.802
Tubulointerstitial fibrosis (%)	10 (5, 17.5) ^b^	12.5 (5, 17.5) ^b^	0.761

eGFR, estimated glomerular filtration rate; a: Data are expressed as means ± standard deviations. b: Data are expressed as medians and interquartile ranges (25% quartile, 75% quartile). c: Data are expressed as frequency (percentages).

### 3.2 Urine proteomics analysis and DEPs

**Figure 1:**
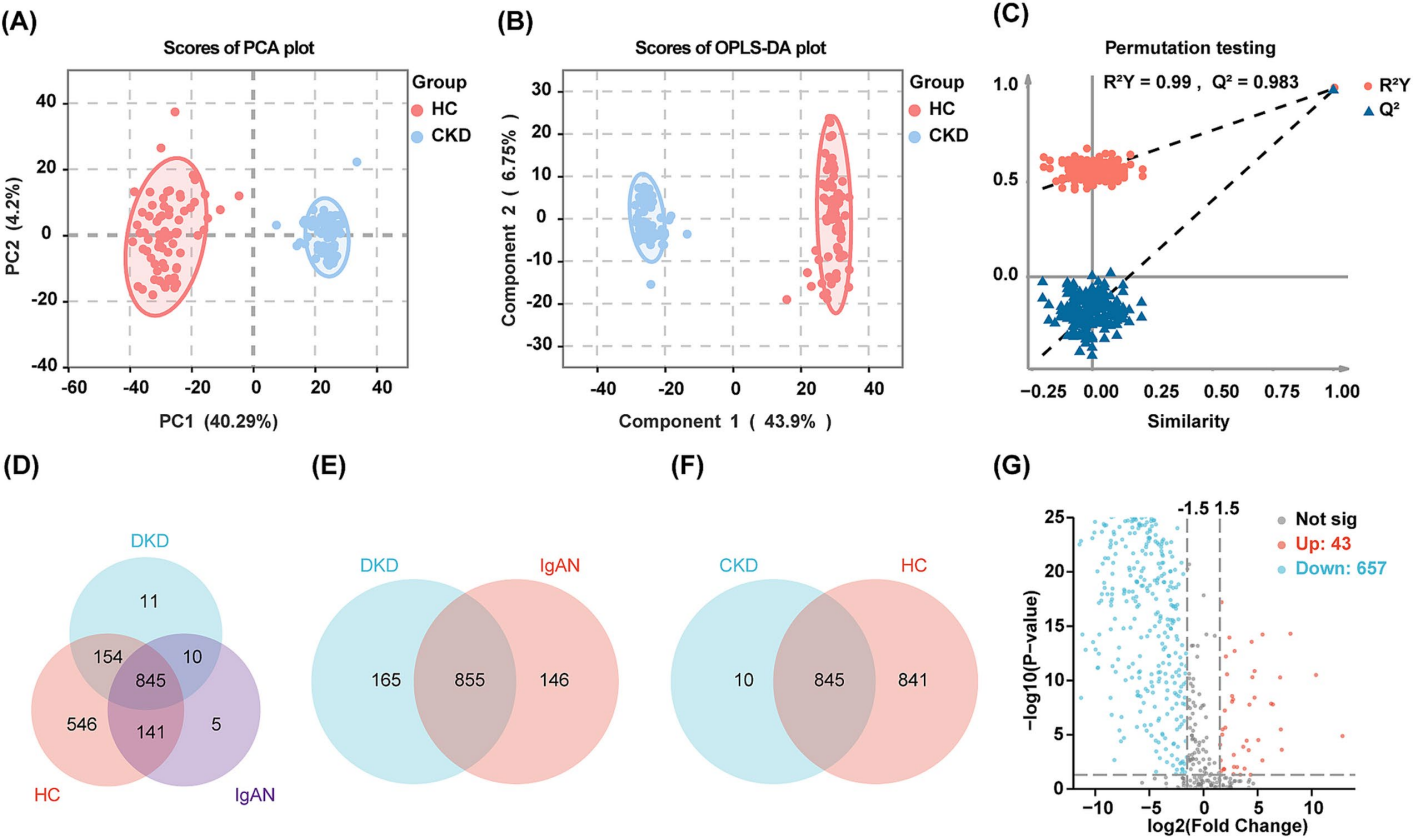
Results of urine proteomics analysis of the discovery cohort. **(A,B)** Two-dimensional scatter plot of PCA and OPLS-DA of urinary proteins in healthy controls and patients with CKD. Dots of the same color represent multiple biologically replicated samples within the same group, and the distribution pattern of the dots reflects the variability between and within groups. The ellipse represents a 95% confidence interval for the samples. **(C)** Orthogonal permutation test plot for OPLS-DA. The horizontal axis shows the permutation retention, which is the proportion of Y variables in the original model that maintain their original order, and the vertical axis shows the values of R^2^ and Q^2^. The red dots and blue triangles represent the statistical data for R^2^ and Q^2^, respectively. The two dashed lines depict the regression lines for R^2^ and Q^2^. The dot marked in the upper right corner represents the R^2^ and Q^2^ values for when the permutation retention is 1. **(D)** The Venn diagram visualizing the overlap of proteins in the HC group and patients with DKD and IgAN. **(E)** The Venn diagram visualizing the overlap of proteins in the patients with DKD and IgAN. **(F)** The Venn diagram visualizing the overlap of proteins in the CKD and HC group. **(G)** Volcano plot of the DEPs between CKD and HC group. Red dots indicate upregulated proteins and blue dots indicate downregulated proteins in the CKD group compared to the HC group.

The PCA score plots demonstrate a distinct separation between the CKD group and the HC group, with components 1 and 2 accounted for 40.29 and 4.2% of the variability, respectively (Figure 1A). Further analysis using OPLS-DA highlighted significant differences in urine proteomics profiles between the HC and CKD groups (Figure 1B). In the orthogonal permutation test for the OPLS-DA model, the R^2^ value was found to be 0.99, while the Q^2^ value was 0.983, indicating a permutation retention equal to 1 (Figure 1C). These results indicated that the system is stable and the urine proteomics profiles of the CKD group were significantly different from those of the HC group.

In total, 1,712 proteins were identified using LC–MS/MS. Specifically, 1,686, 1,020, and 1,001 proteins were identified in the HC, DKD, and IgAN groups, respectively (Figure 1D). For further analyzes within the CKD group, 855 proteins that were common to both the DKD and IgAN groups were utilized (Figure 1E). Additionally, there were 845 proteins that overlapped between the HC and CKD groups (Figure 1F). 700 proteins displayed significant differences, comprising 657 downregulated proteins and 43 upregulated proteins in the CKD group (Figure 1G). Supplementary Figures 1A,B illustrate the DEPs between the DKD and HC groups, as well as between the IgAN and HC groups, respectively.

### 3.3 GO and KEGG enrichment analysis of DEPs

**Figure 2:**
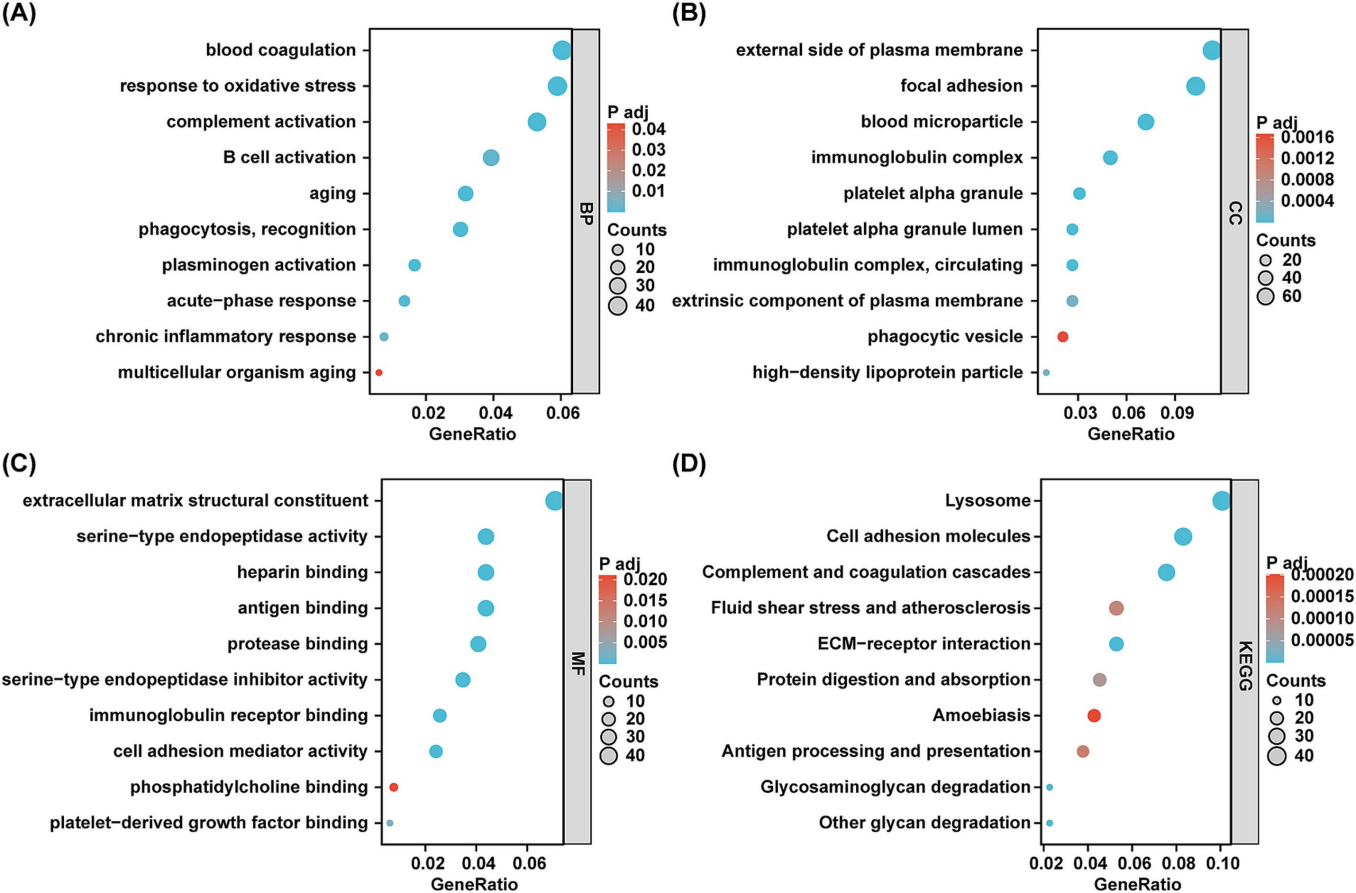
GO terms and KEGG pathways enrichment analyzes of DEPs. **(A–C)** The bubble diagram of GO terms enrichment analyzes. BP, biological processes. CC, cell components. MF, molecular functions. The *x*-axis indicates the gene ratio and the *y*-axis indicates GO terms. Bubble size indicates gene count and color indicates the adjusted *p* value with higher in red and lower in green. **(D)** The bubble plot of KEGG pathway analyzes. The *x*-axis indicates the gene ratio and the *y*-axis indicates KEGG terms. Bubble size indicates gene count and color indicates the adjusted *p* value with higher in red and lower in green.

GO analysis was performed to assess the functional significance of the DEPs. Regarding biological processes, the DEPs were predominantly enriched in pathways associated with oxidative stress response, complement activation, B cell activation, aging, and chronic inflammatory responses—pathways particularly relevant to aging and CKD (Figure 2A). Furthermore, the cellular components and molecular functions of the DEPs were primarily concentrated on immunoglobulin complexes, platelet alpha granules, heparin binding, antigen binding, and immunoglobulin receptor binding (Figures 2B,C). To gain deep insights into the biological roles of the DEPs, we conducted a KEGG enrichment analysis. The three most significant pathways identified were lysosomal function, complement and coagulation cascades, and cell adhesion molecules (Figure 2D). Collectively, the GO and KEGG analyzes corroborated that urinary DEPs are tightly linked to immune responses, complement activation, inflammation, and aging.

### 3.4 Aging-related proteins in the urine from discovery cohort

**Figure 3:**
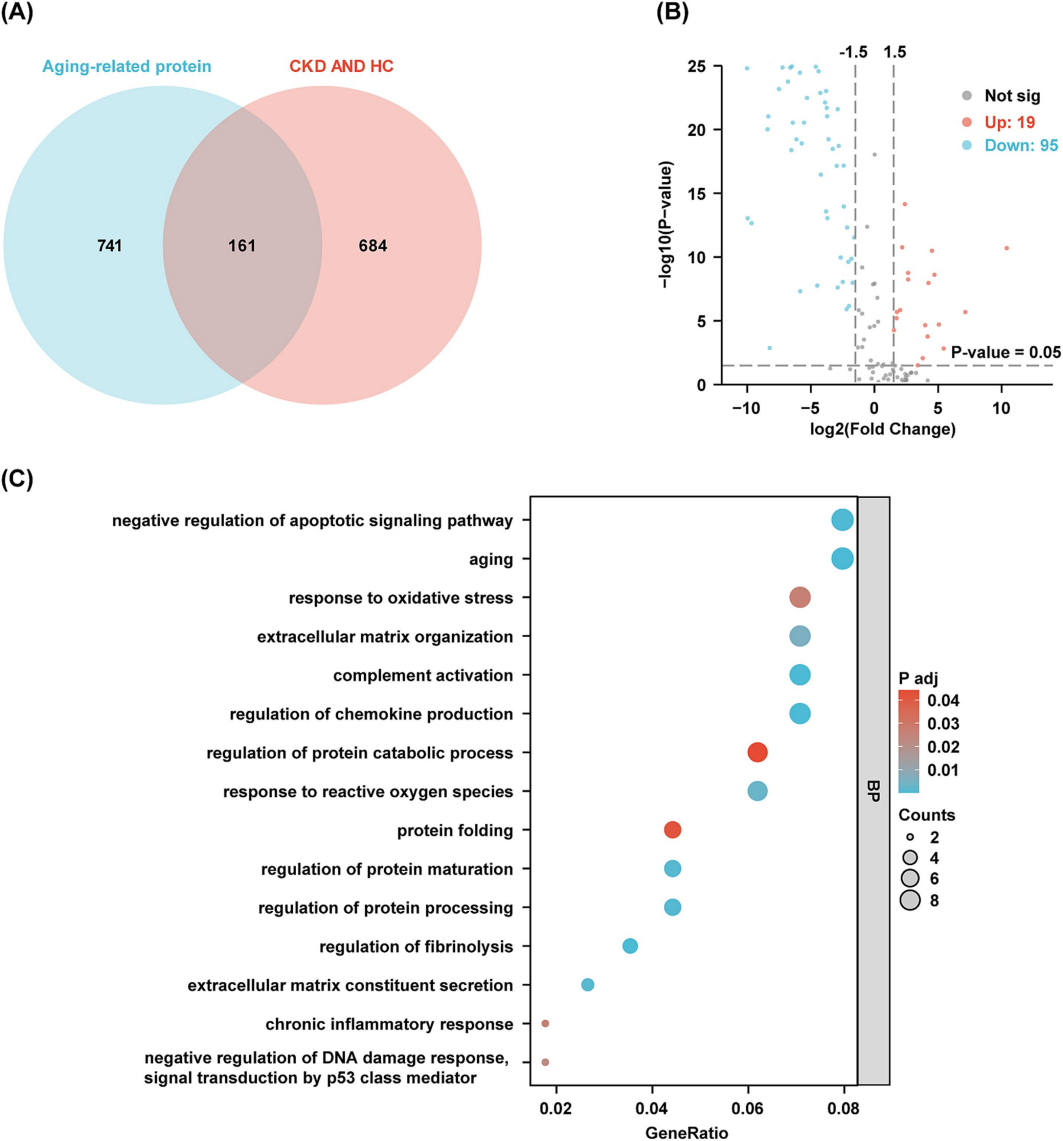
Aging-related proteins in urine and functional analysis. **(A)** The Venn diagram shows the amount of aging-related protein in the urine of discovery cohort. **(B)** Volcano plots visualize the differential aging-related protein in discovery cohort. Red dots indicate proteins up-regulated in the CKD group compared to the HC group, while blue dots indicate proteins down-regulated in the CKD group. **(C)** GO clusters in the enrichment analysis of differentially aging-related protein in urine.

Based on the findings of Oh (18) and Suryadevara (19), a total of 902 candidate protein markers have been identified that characterize aging across 16 distinct organs and tissues. These include adipose tissue, bone marrow, the cardiovascular system, the brain, the immune system, the intestine, kidneys, the liver, lungs, muscle, pancreas, mammary glands, ovaries, placenta, skeleton, and skin. In urine samples, 161 proteins associated with aging were identified (Figure 3A). When comparing the CKD group to the HC group, 114 proteins exhibited significant differences, comprising 95 that were down-regulated and 19 that were up-regulated (Figure 3B). GO and KEGG enrichment analyzes revealed pathways closely linked to the aging process, including aging, chronic inflammatory responses, reactions to reactive oxygen species (ROS), regulation of DNA damage responses, and oxidative stress (Figure 3C). Supplementary Table 1 and Supplementary Figure 1C present aging-related proteins identified in DEPs between the DKD and HC groups. Similarly, Supplementary Table 2 and Supplementary Figure 1D show aging-related proteins in DEPs between the IgAN and HC groups. Collectively, these findings highlight the potential of urinary proteins as biomarkers for aging across various organs and tissues.

### 3.5 Correlation between aging-related proteins and laboratory parameters in patients with CKD in the discovery cohort

**Figure 4:**
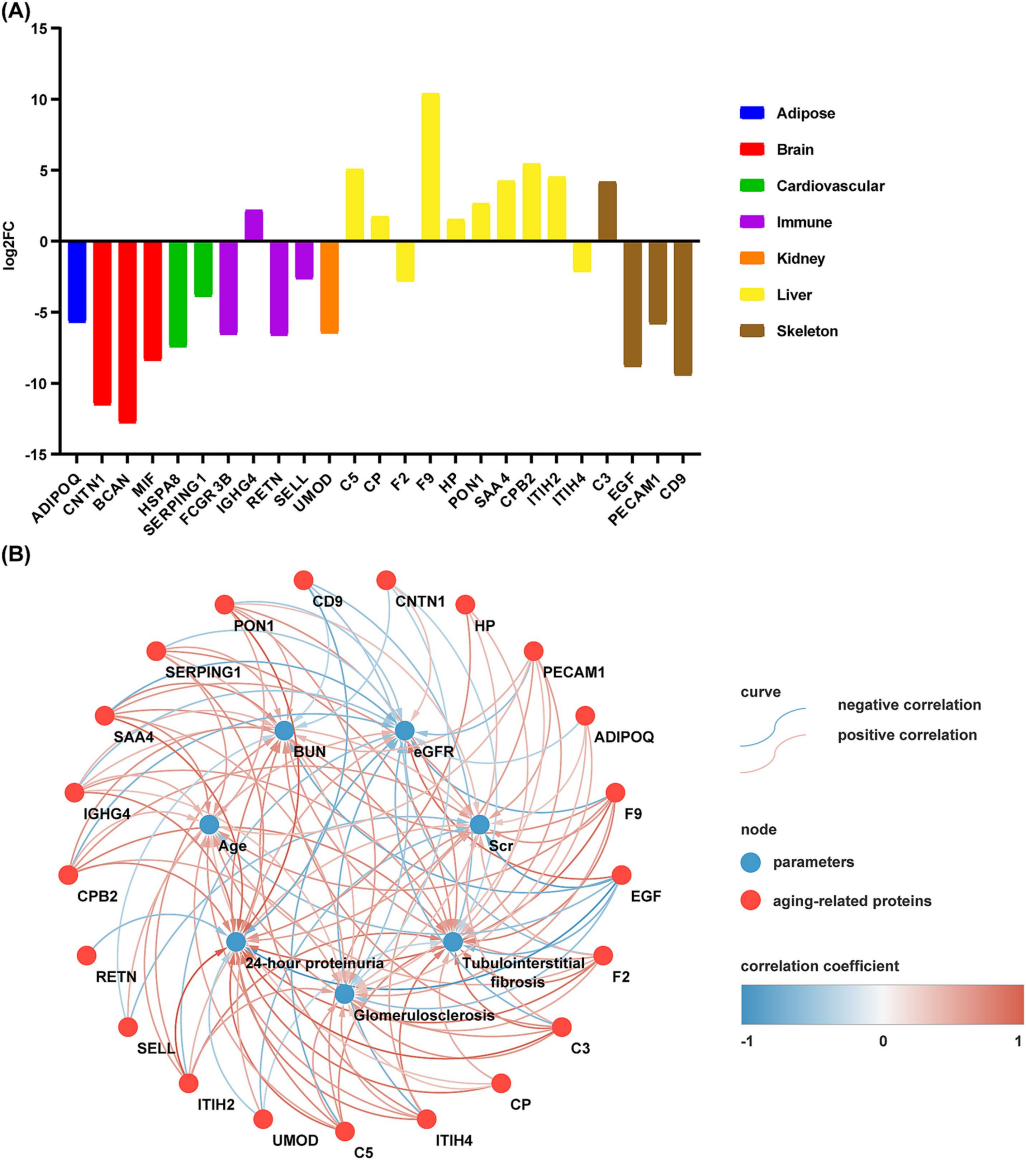
Urinary proteins characterizing aging in specific organs and tissues and their correlation with clinical parameters. **(A)** Proteins in urine that characterize aging in specific organs and tissues. A log_2_FC value of >0 indicates that the protein expression is elevated in the urine of CKD patients compared to controls, and log_2_FC < 0 indicates that protein expression is decreased in the urine of CKD patients compared to HC group. **(B)** Correlation between aging-related protein and clinical parameters in patients with CKD. The red nodes represent aging-related proteins and blue nodes represent clinical parameters. The correlations between these aging-related proteins and clinical parameters are statistically significant (*P* < 0.05).The blue curve represents negative correlation and the red curve represents positive correlation. Darker blue curves indicate a greater degree of negative correlation, while darker red curves indicate a greater degree of positive correlation.

Next, we focused on differentially aging-related protein. After eliminating proteins that exhibited opposing expression patterns in blood or tissue as documented in existing literature, we successfully identified 25 proteins in urine that are linked to aging in specific organs and tissues (Figure 4A). Correlation analysis indicated statistically significant relationships between 21 of these proteins and various factors such as age, BUN, Scr, eGFR, 24-h proteinuria, glomerulosclerosis, and tubulointerstitial fibrosis (*p* < 0.05) (Figure 4B).

Notably, age showed a significant positive correlation with 13 proteins, including plasma protease C1 inhibitor (SERPING1) (= 0.375), serum amyloid A-4 protein (SAA4) (= 0.447), and complement C3 (C3) (= 0.411), while demonstrating a negative correlation with epidermal growth factor (EGF) (= −0.362). BUN was significantly positively associated with 12 proteins, including SERPING1 (= 0.353), complement C5 (C5) (= 0.482) and inter-alpha-trypsin inhibitor heavy chain H4 (ITIH4) (= 0.466), and negatively with 4 proteins, including contactin-1 (CNTN1) (= −0.255), SELL (= −0.241) and EGF (= −0.474). Scr was significantly positively associated with 12 proteins, such as adiponectin (ADIPOQ) (= 0.218), Ig gamma-4 chain C region (IGHG4) (= 0.265), and C5 (= 0.407), and negatively with 3 proteins, including SELL (= −0.365), UMOD (= −0.378), and EGF (= −0.559). eGFR was significantly positively associated with 4 proteins, including SELL (= 0.0.305), UMOD (= 0.325), and EGF (= 0.582), while negatively associated with 13 proteins, including SERPING1 (= −0.293), coagulation factor IX (F9) (= −0.474) and C3 (= −0.415). Additionally, 24-h proteinuria was significantly positively associated with 14 proteins, including SERPING1 (= 0.458), ceruloplasmin (CP) (= 0.57) and C3 (= 0.654), and negatively with 4 proteins, such as UMOD (= −0.286) and EGF (= −0.649).

Interestingly, glomerulosclerosis showed a significant positive correlation with 15 proteins, including SAA4 (= 0.346) and C3 (= 0.435). Conversely, it had a negative association with 3 proteins, such as CNTN1 (= −0.242), EGF (= −0.356), and CD9 (= −0.311). Furthermore, tubulointerstitial fibrosis exhibited a significant positive correlation with 15 proteins, notably C5 (= 0.472) and C3 (= 0.567), while it was negatively associated with 4 proteins, including UMOD (= −0.219), EGF (= −0.442), and the CD9 antigen (CD9) (= −0.347).

### 3.6 Validation of the aging-related protein and clinical parameters in patients with CKD in validation cohort

Patients with CKD often experience associated conditions such as cardiovascular disease, immune dysfunction, and abnormal mineral bone metabolism. To further investigate this, we validated the expression levels of SERPING1, SELL, UMOD, and EGF in urine samples using ELISA within a validation cohort. These markers are potential candidates for characterizing aging in the cardiovascular system, immune system, kidneys, and skeletal system, respectively (18, 19). The levels of SELL, UMOD, and EGF were significantly lower in the CKD group compared to the HC group (*p* < 0.05). However, no statistically significant difference was found for SERPING1 (*p* > 0.05) (see Table 2).

**Table 2:** Differential analysis of aging-related proteins in the urine of subjects from the validation cohort.

Proteins	HC group (*n* = 31)	CKD group (*n* = 51)	*P*- Value
SERPING1 (ng/ml)	936.62 (862.59, 1016.22)	999.36 (675.96, 1099.42)	0.464
SELL (ng/ml)	7.23 (6.57, 7.68)	5.30 (2.86, 7.43)	0.006
UMOD (μg/ml)	37.78 (36.03, 41.85)	31.19 (18.76, 37.74)	<0.001
EGF (pg/ml)	410.66 (367.63, 428.1)	352.19 (253.72, 419.27)	0.007

SERPING1, plasma protease C1 inhibitor; SELL, L-selectin; UMOD, uromodulin; EGF, epidermal growth factor.

Consistent with the findings from urine proteomics in the discovery cohort, a notable negative correlation was observed between age and EGF, as well as between BUN and SELL with EGF. Scr demonstrated a negative association with SELL, UMOD, and EGF, while eGFR showed a positive correlation with SELL, UMOD, and EGF. Additionally, a negative correlation was found between 24-h proteinuria and both UMOD and EGF. Glomerulosclerosis also exhibited a negative association with EGF, and tubulointerstitial fibrosis was negatively correlated with both UMOD and EGF. Furthermore, SELL displayed a significant negative correlation with age, glomerulosclerosis, and tubulointerstitial fibrosis, whereas UMOD was negatively correlated with age, BUN, and glomerulosclerosis. However, in contrast to the discovery cohort, SERPING1 was positively associated with eGFR (see Table 3).

**Table 3:** Correlation between aging-related proteins in urine and clinical parameters in a validation cohort of patients with CKD.

Variable	SERPING1		SELL		UMOD		EGF	
	Rho	*P*-value	Rho	*P*-value	Rho	*P*-value	Rho	*P*-value
Age	−0.148	0.3	−0.325	0.020	−0.373	0.007	−0.463	0.001
Blood urea nitrogen	−0.125	0.381	−0.407	0.003	−0.422	0.002	−0.420	0.002
Serum creatinine	−0.252	0.074	−0.583	<0.001	−0.548	<0.001	−0.563	<0.001
eGFR	0.282	0.045	0.604	<0.001	0.591	<0.001	0.587	<0.001
24-h proteinuria	−0.228	0.107	−0.166	0.245	−0.289	0.039	−0.322	0.021
Glomerulosclerosis	−0.259	0.067	−0.360	0.009	−0.314	0.025	−0.337	0.016
Tubulointerstitial fibrosis	−0.252	0.075	−0.377	0.006	−0.411	0.003	−0.461	0.001

eGFR, estimated glomerular filtration rate; SERPING1, plasma protease C1 inhibitor; SELL, L-selectin; UMOD, uromodulin; EGF, epidermal growth factor.

## 4 Discussion

In this study, we employed LC–MS/MS-based urine proteomics to identify potential aging biomarkers in the urine of patients with CKD. The results from PCA and OPLS-DA revealed significant changes in the urinary protein profiles of CKD patients. In the discovery cohort, we identified several proteins that were significantly upregulated, including haptoglobin (HP), alpha-1-antitrypsin (SERPINA1), alpha-2-macroglobulin (A2M), SAA4, IGHG4, C3, C5, complement component C8 alpha chain (C8A), complement component C8 gamma chain (C8G), complement component C9 (C9), and carboxypeptidase B2 (CPB2). These proteins are implicated in the regulation of the acute phase response, B cell activation, and complement activation. Notably, similar abnormalities have been observed in kidney transplant recipients experiencing acute rejection and BK virus infection, indicating that CKD may cause significant immune dysfunction (26, 27). Moreover, among the 657 down-regulated proteins identified, tetranectin (CLEC3B), protein AMBP, ephrin type-B receptor 6 (EPHB6), and vesicular integral-membrane protein VIP36 (LMAN2) have been linked to a decreased risk of eGFR decline in patients with CKD (28). As these proteins may serve as protective factors that slow the progression of CKD, investigating their relationship with aging in this patient population could yield valuable insights.

We identified 114 DEPs linked to the aging process. A functional enrichment analysis of these DEPs reveals critical pathways associated with the aging process, including the aging, responses to ROS and oxidative stress, as well as the DNA damage response. It is well known that oxidative stress impairs cellular structure and function, and ROS-induced oxidative stress accelerates cellular senescence by inducing DNA and lipid peroxidation damage, promoting telomere shortening, and inhibiting protein degradation and repair (29, 30). Mitochondria are the main source of ROS, and mitochondrial quality control (MQC) keeps ROS at a relatively low and stable level. The organism’s ability to scavenge ROS decreases during aging. Excessive ROS induces oxidative stress leading to mitochondrial DNA mutations and protein oxidation. Abnormal mitochondrial products ultimately lead to reduced mitochondrial biosynthesis and MQC imbalance, which exacerbates oxidative stress and creates a vicious cycle that accelerates aging (31).

Furthermore, DNA damage is a crucial factor in the aging process, contributing to genomic instability through mutations and chromosomal abnormalities. It significantly impacts the depletion of stem cell reserves and disrupts intercellular communication by triggering apoptosis, cellular senescence, and premature differentiation of stem cells, along with reduced growth signaling (32). Additionally, we found that the functions of these DEPs are linked to protein folding, processing, and maturation, as well as protein catabolism, all of which are essential for maintaining protein homeostasis. Overall, our research reveals that a variety of aging-related proteins, which influence different facets of the aging process, can be identified in the urine of patients with CKD.

SELL, also known as CD62L, is a type-I transmembrane glycoprotein and cell adhesion molecule that plays an important role in modulating immune cell trafficking. Recently, SELL has been recognized as a marker of immune senescence (25). T cell subsets experience considerable changes with aging. This is reflected in a decline of naive T cells, which are marked by CD62L expression, and a rise in memory-like T cells, indicated by the loss of CD62L and an increase in CD44 expression (33).

Neutrophils are important effector cells of innate immunity, and CD62 expression is reduced in aging neutrophils. Several studies have revealed that an increase in aging neutrophilia is associated with aging-related diseases such as stroke and atherosclerosis (34, 35). Aging neutrophils exhibit high levels of mitochondrial ROS and mitochondrial DNA leakage that promote neighboring cellular senescence (36). In addition, Neutrophils can induce telomere dysfunction via paracrine ROS and stimulate neighboring cells to secrete senescence-associated secretory phenotype cytokines, which ultimately contribute to organ dysfunction in aging and disease (37). For circulating levels of SELL, serum SELL was reduced in patients with ESKD compared to healthy subjects (38). Consistently, we observed relatively low levels of urinary SELL (uSELL) in patients with CKD. Notably, uSELL exhibited a significant positive correlation with eGFR and a negative correlation with factors such as age, glomerulosclerosis, and tubulointerstitial fibrosis. Furthermore, uSELL was positively associated with renal disease activity in patients with lupus nephritis, and low uSELL levels emerged as an independent predictor of a high chronicity index (39). The evidence presented indicates that low levels of uSELL are indicative of immune system aging in patients with CKD.

UMOD is exclusively produced by the kidneys. Elevated urinary UMOD (uUMOD) levels are indicative of better kidney tubular health, and these higher levels are associated with a decreased severity of tubular atrophy and interstitial fibrosis (40, 41). Furthermore, in diabetic patients with eGFR <60 mL/min per 1.73 m^2^ and in patients with CKD, higher uUMOD reduced the risk of progression to ESKD and cardiovascular death (42, 43). The anti-inflammatory and systemic antioxidant properties of uUMOD may help elucidate its association with cardiovascular events and overall renal outcomes (44, 45). Conversely, low levels of uUMOD were found to be an independent predictor of rapid decline in eGFR and progression to ESKD in patients with CKD, and uUMOD concentration showed a significant positive correlation with eGFR and a negative correlation with proteinuria, which is consistent with our findings (46). In summary, uUMOD serves as a significant biomarker for assessing kidney aging, with lower levels indicating diminished functional reserve.

The concentration of urinary EGF (uEGF) declines with age as previously reported (47, 48). We found that uEGF was negatively correlated with age in patients with CKD. In addition, uEGF is reduced in a variety of kidney diseases (49, 50). The lower uEGF was strongly associated with a more rapid eGFR decline, and low uEGF is a potential marker for the progression of CKD, including DKD (51) and IgAN (52). We also observed a significant positive correlation of uEGF with eGFR and a significant negative correlation with glomerulosclerosis and tubulointerstitial fibrosis, which is consistent with previous report (53). Thus, higher uEGF is considered a protective factor. It is worth mentioning that EGF has been recognized as a marker of skeleton aging in recent study, although the underlying mechanism remains a mystery (19). Given that patients with CKD are often complicated by abnormal bone metabolism, especially when combined with osteoporosis, we believe that uEGF might be a promising new addition to the list of skeletal aging markers in CKD-based populations.

As a member of the serpin family of protease inhibitors, previous studies have shown that SERPING1 is associated with cardiovascular aging and hereditary angioedema (19, 54). In addition, SERPING1 protein levels were reduced in the blood of patients with restless legs syndrome (55). The concentration of urinary SERPING1 was also decreased in older adults with chronic diseases compared to age-matched healthy populations (56). Similar to these results, we found reduced levels of SERPING1 in the urine of patients with CKD in discovery cohorts. However, the correlation between SERPING1 and eGFR was not consistent across the discovery and validation cohorts. SERPING1 is a gene encoding a C1- esterase inhibitor protein. It is reported that the gene expression of SERPING1 in renal tubules of patients with DKD is negatively related to GFR, which is consistent with the correlation observed in the discovery cohort (57). However, in a randomized, double-blind, placebo-controlled study, the decline in eGFR levels was prevented by treatment with C1 esterase inhibitors in renal transplant patients (58). This study supports that SERPING1 may contribute to the maintenance of renal function, potentially suggesting the positive correlation between SERPING1 and eGFR that we observed in the validation cohort. Indeed, the correlation between urinary SERPING1 and eGFR is unclear based on the available studies. Therefore, the inconsistent correlation observed in the discovery cohort and the verification cohort may be related to the small sample size. Expanding the sample size and including more types of kidney disease patients in future studies could help to further reveal the association between urinary SERPING1 and eGFR.

ADIPOQ is an endogenous cytokine secreted primarily by adipose tissue. We found decreased expression of ADIPOQ in patients with CKD. ADIPOQ deficiency leads to impaired glucose metabolism and lipid homeostasis, and promotes inflammation and fibrosis in multiple tissues, resulting in reduced healthspan (59). ADIPOQ treatment restores *β*-cell functional integrity and glucose metabolism and improves cognitive function to some extent (60, 61).Thus, the ability to prolong healthspan by maintaining ADIPOQ levels provides a promising therapy for aging-related diseases. CNTN1 is an axonal protein commonly associated with autoimmune neuropathies (62). The plasma CNTN1 levels were an independent protective factor for late-life prefrailty and frailty (63). Recently, CNTN1 has been reported as a novel target antigen for membranous nephropathy (63). These new insights into cross-systems syndromes should facilitate earlier diagnosis and more timely use of effective treatment to delay aging.

Among the proteins characterizing liver aging, serum levels of C5 increase with age in healthy caucasians population (64). In this article, we found increased levels of C5 in the urine of patients with CKD compared to the HC group, which potentially suggests that CKD promotes aging. Complement activation leads to the release of complement component C5a in an ischemia/reperfusion-induced acute kidney injury model. C5a activates Wnt4/*β*catenin signaling by inducing aberrant DNA methylation modifications in renal tubular epithelial cells, which in turn accelerates tubular senescence. Both inhibition of C5a receptor 1 (C5aR1) and inhibition of the Wnt4/β-catenin signaling pathway via C1 inhibitors attenuate tubular senescence (65). C5a also mediates cellular senescence in diabetic nephropathy, and the C5aR1 inhibitor PMX53 attenuates cellular senescence (66). Thus, complement inhibitors may represent a novel therapeutic strategy to delay cellular senescence in patients with kidney disease. In addition, increased expression of CP, F9, and HP was observed in the kidney tissues of mice treated with calcium oxalate crystals (67). All these represent areas of great interest for future research on these multifaceted proteins.

One of the strengths of our study is that all patients with CKD underwent renal biopsy, which offers robust evidence for characterizing renal aging. The study faced several limitations. Firstly, the sample size was relatively small. Secondly, the specific populations of DKD and IgAN limit the generalizability of our findings to the broader CKD population; however, they do represent a significant portion of nephrology patients. Thirdly, as this was a cross-sectional study, we cannot establish causal relationships between age-related proteins and clinical parameters. Conducting longitudinal follow-up studies to monitor changes in urinary age-related proteins as CKD progresses could provide more clinically relevant insights. Lastly, we did not collect data on other potential confounding factors, such as lifestyle, medication use, and comorbidities beyond hypertension and diabetes, all of which may influence the study outcomes. The fact that all the patients with CKD were newly diagnosed and therefore had not been received any immunosuppressive treatment decreased possible treatment-related bias. Future studies with longitudinal designs, larger sample sizes, more diverse range of CKD types and more comprehensive data collection are necessary to address these limitations, and provide a more comprehensive understanding of aging process in patients with CKD.

## 5 Conclusion

In conclusion, this study emphasizes the promise of LC–MS/MS-based urine proteomics analysis in identifying protein markers that characterize aging in specific organs and tissues of patients with CKD. Notably, SELL, UMOD, and EGF have been recognized as potential markers indicative of aging. Once validated in future studies, these candidate urinary biomarkers of aging could be utilized more widely to characterize organ and tissue aging associated with CKD, thereby enhancing clinical monitoring and optimizing the overall management of the disease.

## Data Availability

The datasets presented in this study can be found in online repositories. The names of the repository/repositories and accession number(s) can be found below: http://www.proteomexchange.org/, PXD018996.
