# Supplementary figures and images for: Proteomics analysis reveals age-related proteins in the urine of chronic kidney disease patients
Source: Front Med (Lausanne). 2025 Jan 6;11:1506134. doi: 10.3389/fmed.2024.1506134 (PMC11743183; doi:10.3389/fmed.2024.1506134)

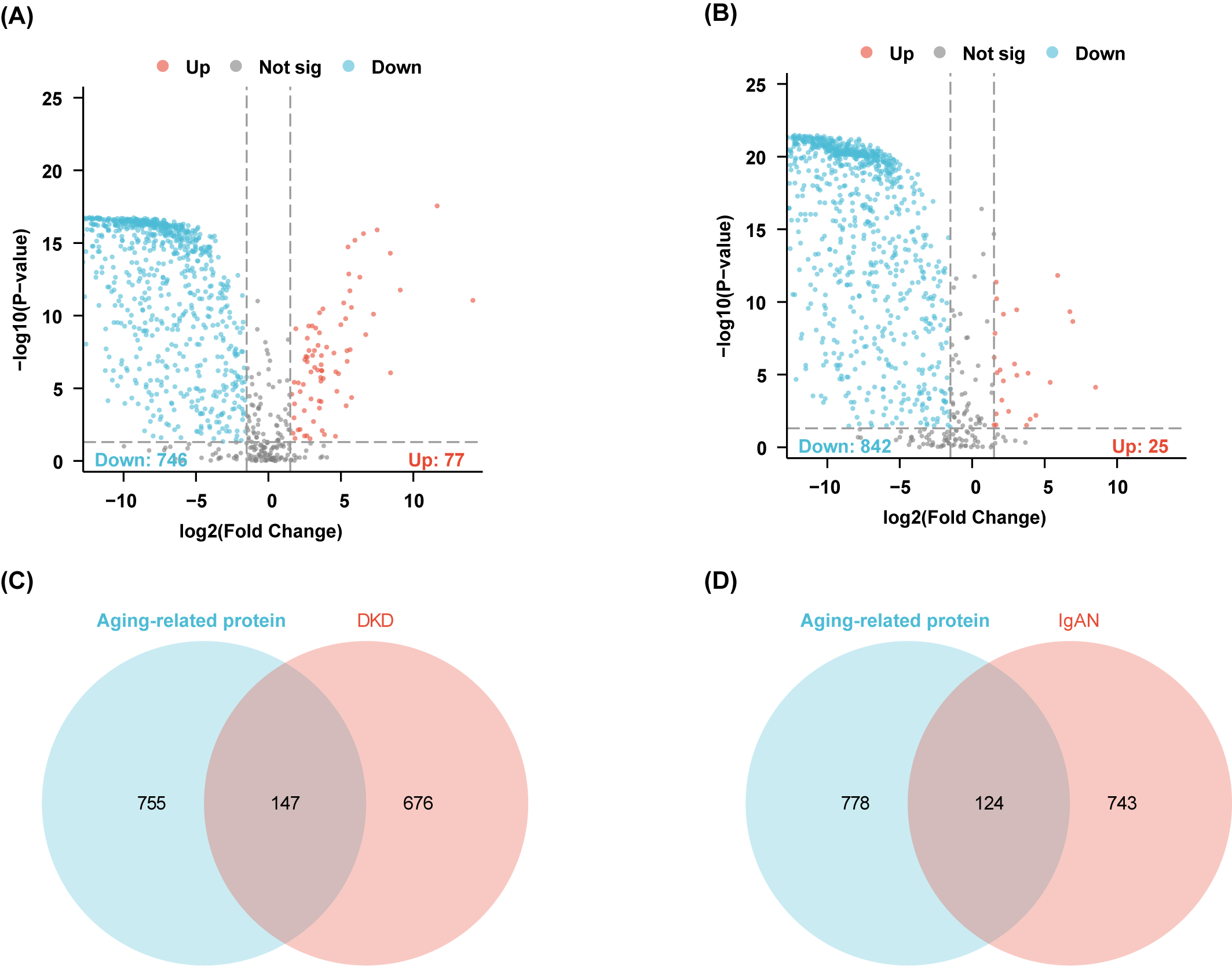

Supplement: Supplementary file 1 [file Image_1.TIF]
